# Ubiquitin C-terminal hydrolase L1 (UCHL1) regulates post-myocardial infarction cardiac fibrosis through glucose-regulated protein of 78 kDa (GRP78)

**DOI:** 10.1038/s41598-020-67746-4

**Published:** 2020-06-30

**Authors:** Qian Lei, Tao Yi, Hang Li, Zhijie Yan, Zhan Lv, Gerui Li, Yanggan Wang

**Affiliations:** 1 Department of Cardiology, Zhongnan Hospital of Wuhan University, Wuhan University, Wuhan, China; 2 Department of Internal Medicine, Zhongnan Hospital of Wuhan University, Wuhan University, Wuhan, 430071 China; 3 Medical Research Institute of Wuhan University, Wuhan University, Wuhan, China; 4 Department of Cardiology, Zhongshan People’s Hospital, Zhongshan, China

**Keywords:** Cardiology, Cardiovascular diseases

## Abstract

Abnormal cardiac fibrosis indicates cardiac dysfunction and poor prognosis in myocardial infarction (MI) patients. Many studies have demonstrated that the ubiquitin proteasome system (UPS) plays a significant role in the pathogenesis of fibrosis. Ubiquitin C-terminal hydrolase L1 (UCHL1), a member of the UPS, is related to fibrosis in several heart diseases. However, whether UCHL1 regulates cardiac fibrosis following MI has yet to be determined. In the present study, we found that UCHL1 was dramatically increased in infarct hearts and TGF-β1-stimulated cardiac fibroblasts (CFs). Inhibition of UCHL1 with LDN57444 (LDN) reversed the myocardial fibrosis in post-MI heart and improved cardiac function. Treatment of LDN or UCHL1 siRNA abolished the TGF-β1-induced fibrotic response of CFs. We further identified GRP78 as an interactor of UCHL1 through screening using immunoprecipitation-mass spectrometer. We determined that UCHL1 interacted with glucose-regulated protein of 78 kDa (GRP78) and prompted GRP78 degradation via ubiquitination. Furthermore, we found that GRP78 was upregulated after UCHL1 knockdown and that the GRP78 inhibitor HA15 diminished the antifibrotic function exerted by UCHL1 knockdown in CFs stimulated with TGF-β1. This suggests that UCHL1 regulates cardiac fibrosis post MI through interactions with GRP78. This work identifies that the UCHL1-GRP78 axis is involved in cardiac fibrosis after MI.

## Introduction

Myocardial infarction (MI) has been the main cause of cardiovascular diseases for centuries and remains a major issue. Although improved survival from acute MI has been observed due to the effectiveness of revascularisation and other therapies, the incidence of heart failure has increased as a consequence of adverse ventricular remodelling^1,2^. Among the factors involved in ventricular remodelling, cardiac fibrosis, which results from the disequilibrium of synthesis and deregulation of extracellular matrix, is a pivotal^3,4^. In the acute stage, cardiac fibrosis, is a repairing process that protects the infarct heart from rupture; at the subacute and chronic stage, in cases where cardiac fibrosis abnormally persists, it inevitably leads to cardiac dysfunction and ventricular wall stiffness increasing the risk of heart failure^4,5^. Thus, it is essential to control levels of cardiac fibrosis.

Cardiac fibroblasts (CFs) are central mediators of the cardiac fibrotic response^6^. CFs are mainly stimulated by TGFβ-1 following MI and differentiate into activated myofibroblasts which express α-smooth muscle actin (α-SMA). This results in the secretion of a large amount of extracellular matrix, including fibronectin and collagen I (Col1), which is, in part, regulated by the activation of Smad2/3^4,7,8^. To date, no studies have fully elucidated how this process is regulated. The ubiquitin proteasome system (UPS), which consists of E1 ubiquitin-activating enzymes, E2 conjugating enzymes and E3 ubiquitin ligases and deubiquitinating enzymes, is responsible for the degradation and stability of the majority of proteins^9,10^. Recently, the UPS has been shown to be essential in the differentiation of various cell types, including induced pluripotent stem cells, adipocytes and lung fibroblasts. Interference in the UPS leads to deficits in differentiation^11-13^. In addition, the UPS is fundamental in controlling cardiac fibrosis in cardiac hypertrophy^14,15^. To investigate how the UPS regulates CF activation under the conditions of MI, we focused on ubiquitin C-terminal hydrolase L1 (UCHL1), a member of UPS family.

UCHL1, which was initially thought to be a neuronal marker, was found to be expressed in the other tissues, such as heart, liver and kidney, among others^16^. Previous studies have shown that the role of UCHL1 varies in a context-dependent manner. UCHL1 can function as an ubiquitin ligase in its dimer form other than hydrolase^17^, UCHL1 also stabilises proteins via targeting glutathione^18,19^. Therefore, there is a dual action of UCHL1 in a variety of diseases, including kidney diseases, neurodegenerative diseases and cancers, with UCHL1 affecting cellular proliferation, cell cycle, migration and invasion^20-25^. To date, only a few studies have assessed the role of UCHL1 in heart diseases. One study found that UCHL1 was markedly upregulated in post-MI heart^26^ and another suggested that UCHL1 acts as a regulator of the Ang II-induced atrial fibrillation and inhibition of it attenuated atrial fibrosis in vivo^27^. Inspired by the finding that the UCHL1 exerts both endogenous and exogenous pro-activation effects in hepatic stellate cells, which are a type of fibroblast^28,29^, we hypothesised that UCHL1 may promote cardiac fibrosis following MI via induction of CF activation.

In this study, we investigated the role of UCHL1 in mouse MI models and primary CFs. Of note, we found that inhibition of UCHL1 improved cardiac function and attenuated cardiac fibrosis post MI. Furthermore, we found that inhibition and knockdown of UCHL1 hindered the cardiac fibrotic response via upregulation of glucose-regulated protein of 78 kDa (GRP78) in CFs. The underlying mechanism of this is largely attributed to the interaction between UCHL1 and GRP78, and the subsequent degradation of GRP78 by ubiquitination. Therefore, we developed a novel mechanism for maladaptive cardiac fibrosis post MI, providing insight into potential pathways to target for novel antifibrotic therapies.

## Results

### UCHL1 is elevated in post MI hearts and CFs treated with TGF-β1

**Figure 1:**
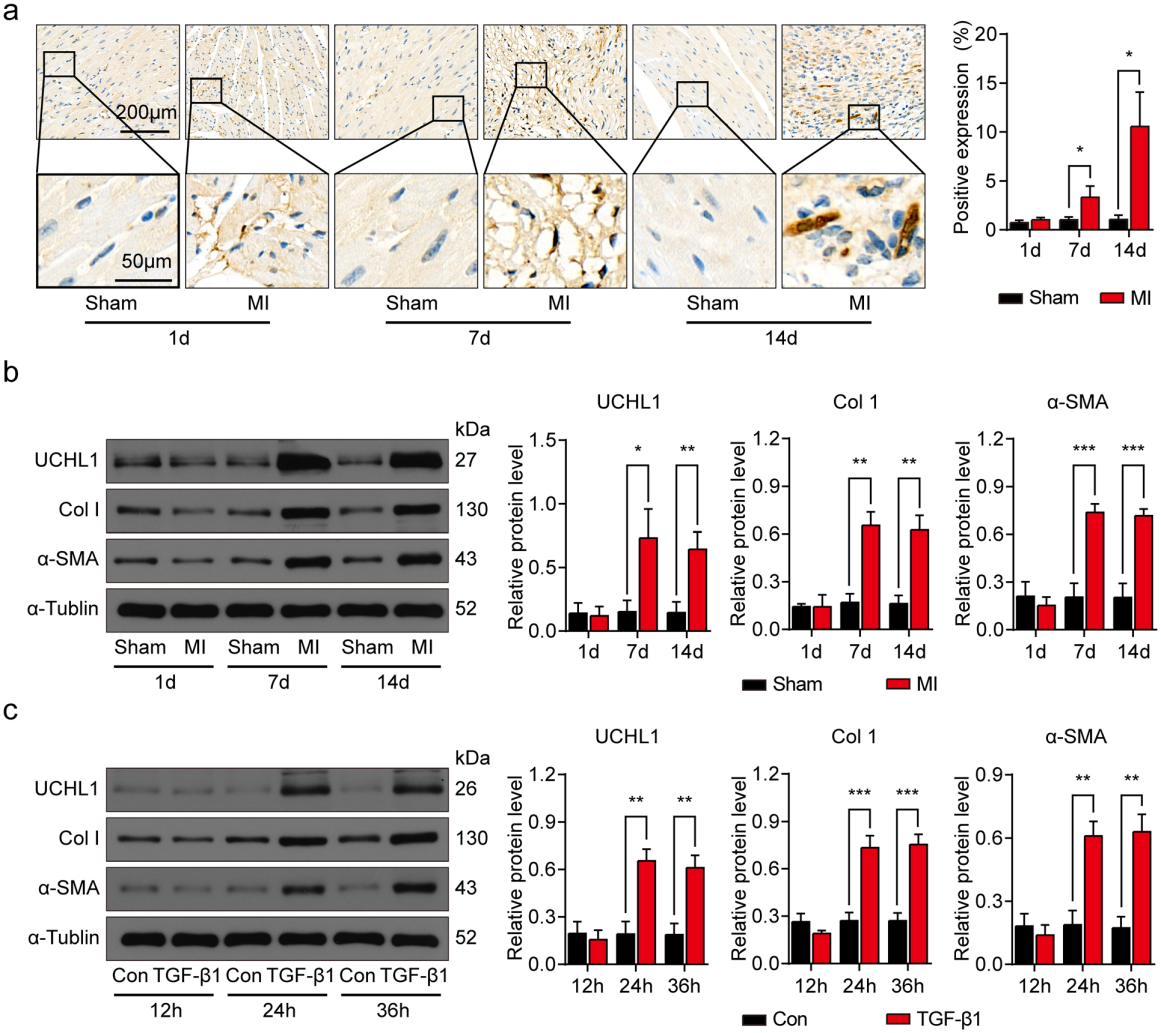
UCHL1 is upregulated in post MI mouse hearts and CFs treated with TGF-β1. (**a**) IHC showing the expression of UCHL1 in sham and MI hearts at 1, 7 and 14 days after operation. n = 3, *P < 0.05. Scale bar (upper) = 200 μm. Scale bar (lower) = 50 μm. (**b**) Western blot and average data showing UCHL1, Col1 and α-SMA protein levels in hearts of sham and MI at 1, 7 and 14 days after operation. n = 3, *P < 0.05, **P < 0.01, ***P < 0.001. (**c**) Western blot and average data showing UCHL1, Col1 and α-SMA protein levels in CFs treated with and without TGF-β1 for indicated times. n = 3, **P < 0.01, ***P < 0.001.

To determine if UCHL1 regulates cardiac fibrosis, we measured UCHL1 expression in post-MI hearts. IHC showed that UCHL1 was highly expressed in the infarct area of MI hearts at 7 days and 14 days after MI as compared with corresponding areas of sham hearts (Fig. 1a). Importantly, the protein expression level of UCHL1 was increased at 7 and 14 days post MI. There were also increased levels of pro-fibrotic proteins, Col1 and α-SMA (Fig. 1b), indicating that UCHL1 was enhanced at 7 days post-MI, and that the increased levels of UCHL1 last at least 7 days.

Fibrotic models were established in CFs with TGF-β1 stimulation. UCHL1 expression in CFs was significantly increased at 24 and 36 h after TGF-β1 (10 ng/ml) stimulation, compared with cells without TGF-β1 stimulation (Fig. 1c). Col1 and α-SMA were also increased accordingly (Fig. 1c), indicating that TGF-β1 induces fibrotic response in CFs. Taken together, our data show that UCHL1 protein levels increased in both fibrotic post-MI hearts and TGF-β1-induced CFs, suggesting that UCHL1 may be involved in the process of cardiac fibrosis following MI.

### Inhibition of UCHL1 by LDN mitigated post-MI fibrosis and enhanced cardiac function

**Figure 2:**
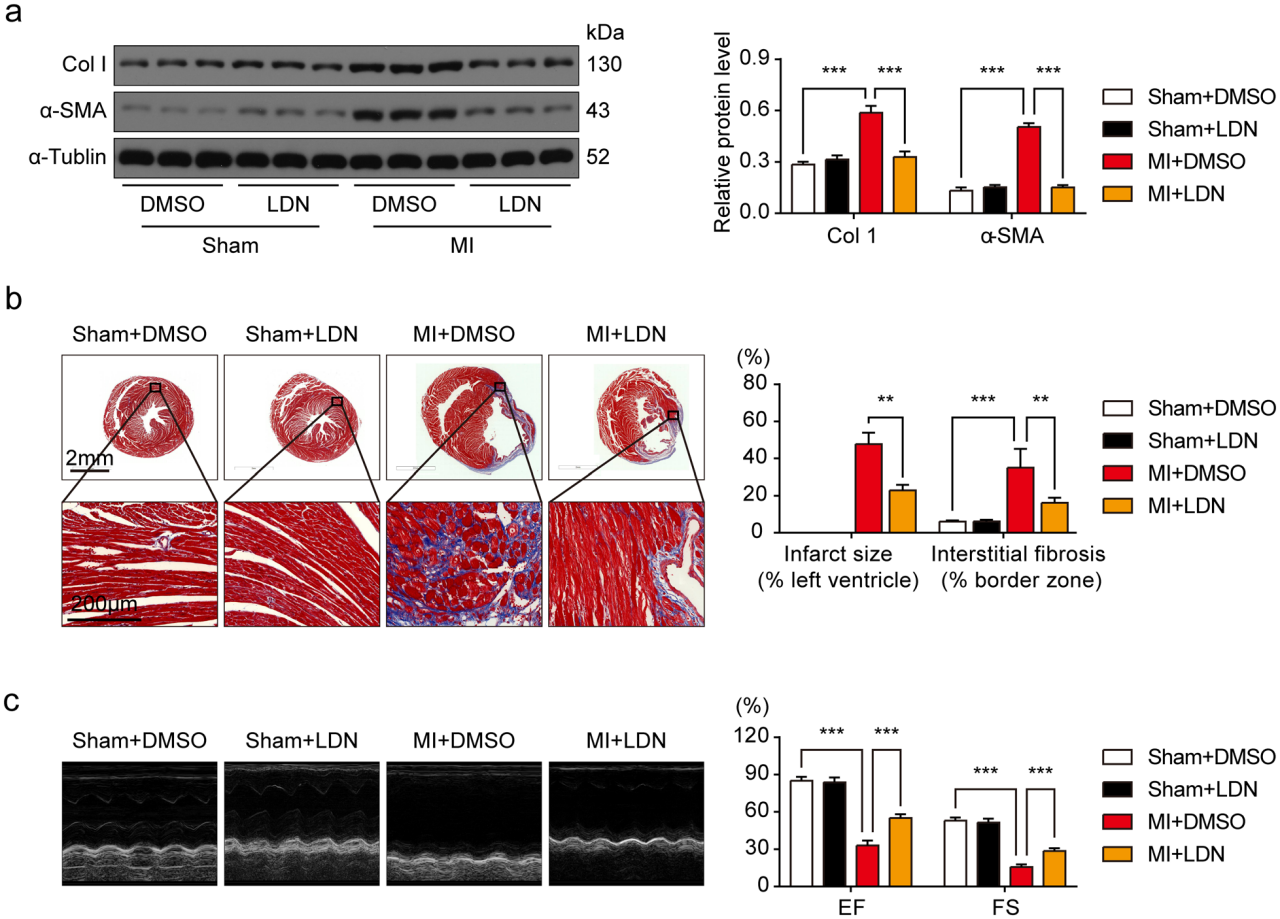
Inhibition of UCHL1 by LDN alleviated post-MI fibrosis and improved cardiac function. (**a**) Western blot and average data showing Col1 and α-SMA protein levels in sham and MI hearts with or without LDN treatment. (**b**) Masson trichrome staining showing infarct size of heart and collagen deposition in the MI cardiac border zone. n = 3. Scale bar (upper) = 2 mm, Scale bar (lower) = 200 μm. (**c**) Representative M-mode images of echocardiography and average data of EF and FS. n = 6. **P < 0.01, ***P < 0.001.

To assess whether UCHL1 mediates post-MI cardiac fibrosis in vivo, we used LDN to inhibit UCHL1. As LDN can also inhibit UCHL3, we determined the UCHL3 protein level in the sham group and MI group 14 days after operation and found that no significant differences between the two groups (Supplemental Fig. 1). Profibrogenic proteins, α-SMA and Col1, significantly increased in hearts 14 days after MI, as compared with the sham hearts (Fig. 2a). Fibrotic factors were significantly reduced by LDN in the hearts of the MI group at 14 days (Fig. 2a). Staining of collagen deposition by Masson's trichrome staining in sham/MI hearts and with/without LDN treatment showed that the UCHL1 inhibitor LDN dramatically prevented MI-associated infarct size and cardiac fibrosis (Fig. 2b). Our data indicate that UCHL1 inhibition prevents the heart from post-MI fibrosis. As cardiac fibrosis is a major factor in post-MI cardiac dysfunction, we next measured cardiac function in sham/MI mice treated with/without LDN. Echocardiography results showed that cardiac ejection fraction (EF) and fractional shortening (FS) in MI mice was significantly reduced at 14 days after MI as compared with sham mice (Fig. 2c). However, UCHL1 inhibitor, LDN, significantly improved post-MI cardiac function. Our data supports the hypothesis that UCHL1 inhibition has a protective effect on cardiac function after MI.

### Both inhibition of UCHL1 by LDN and knockdown UCHL1 by siRNA downregulated TGF-β1-mediated expression of pro-fibrotic proteins in CFs

**Figure 3:**
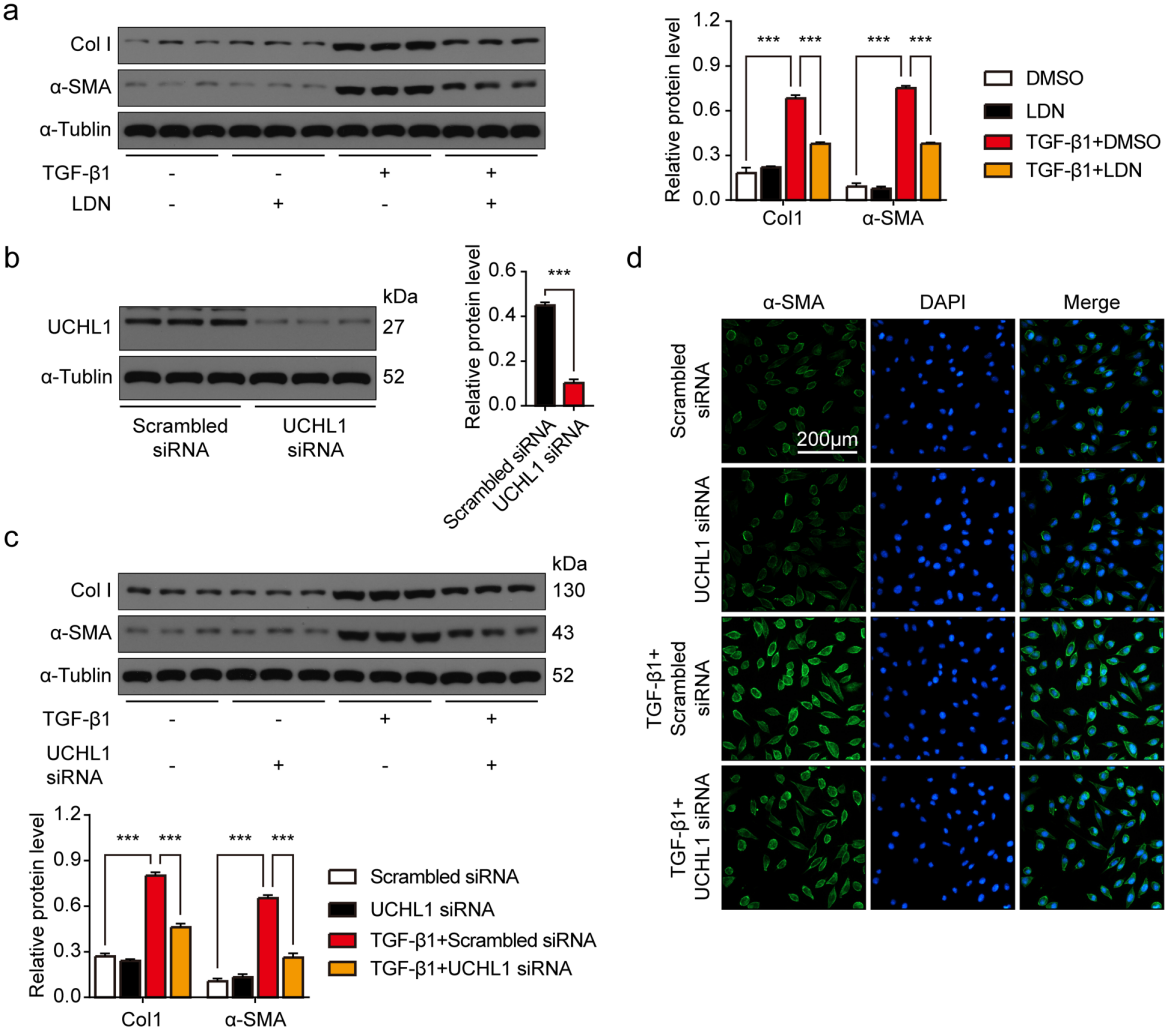
Both inhibition and knockdown of UCHL1 downregulated TGF-β1-mediated expression of pro-fibrotic proteins in CFs. (**a**) Western blot and average data showing Col1 and α-SMA protein levels in CFs treated with/without LDN with/without TGF-β1 stimulation. n = 3, ***P < 0.001. (**b**) Western blot and average data showing UCHL1 protein level in CFs with/without UCHL1 knockdown. n = 3, ***P < 0.001. (**c**)Western blot and average data showing Col1 and α-SMA protein levels in CFs with/without UCHL1 knockdown treated with/without TGF-β1 stimulation. n = 3, ***P < 0.001. (**d**) Immunofluorescence staining of α-SMA in CFs with/without UCHL1 knockdown treated with/without TGF-β1 stimulation. Scale bar = 200 μm.

We next suppressed activity of UCHL1 in CFs with LDN. While TGF-β1 stimulation increased profibrogenic proteins Col1 and α-SMA in CFs, inhibition of UCHL1 blocked TGF-β1-induced increases of these profibrogenic proteins (Fig. 3a). We next used siRNA to knock down UCHL1 in CFs (Fig. 3b). We found that UCHL1 siRNA decreased TGF-β1-induced increases of these profibrogenic proteins (Fig. 3c). The UCHL1 siRNA preventing CFs from fibrosis induced by TGF-β1 was also observed by immunofluorescence staining with α-SMA antibody (Fig. 3d). Collectively, our results show that UCHL1 regulates the fibrotic response of CFs.

### UCHL1 regulated fibrotic responses through GRP78

**Figure 4:**
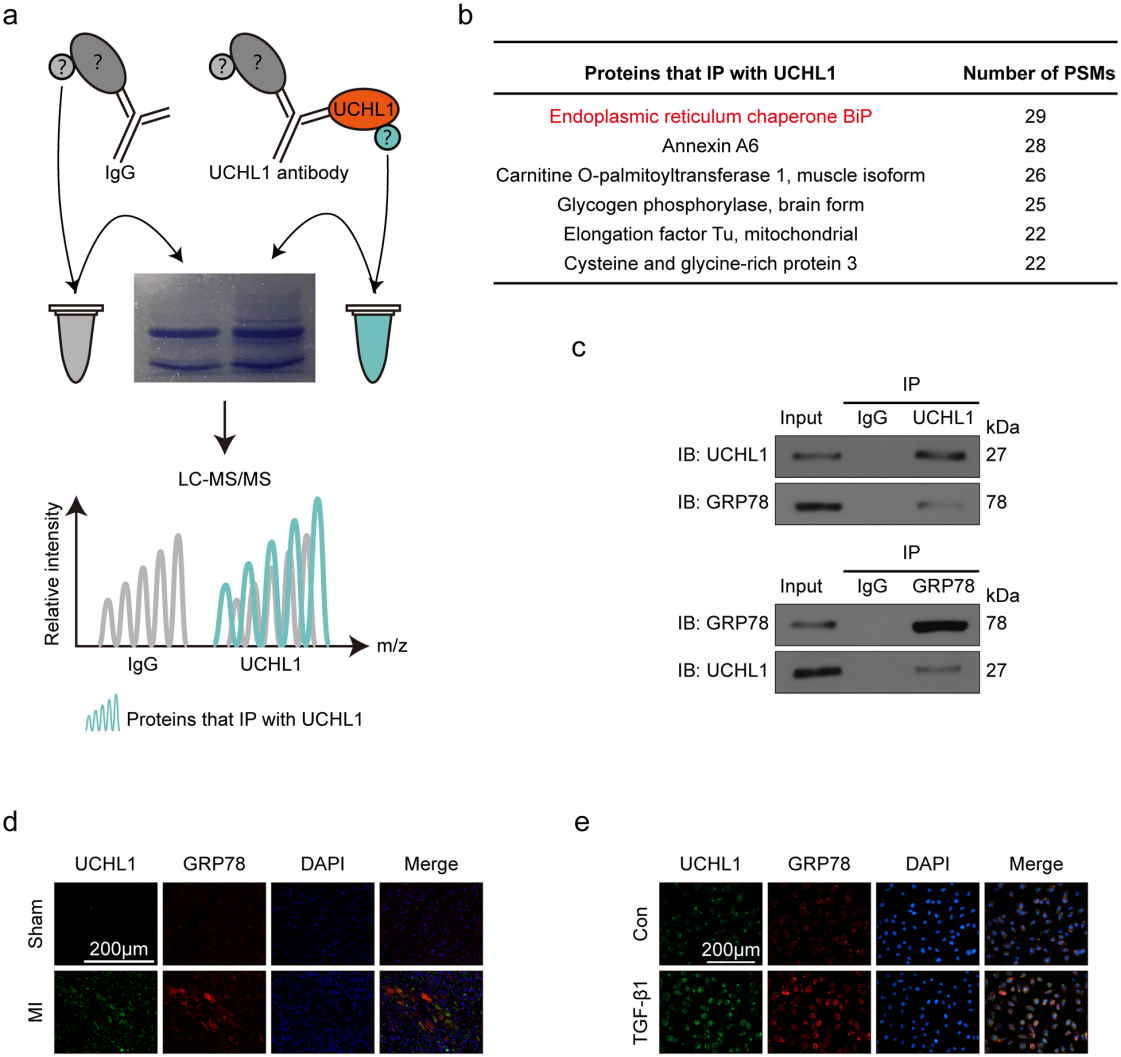
UCHL1 interacts with GRP78. (**a**) Schematic depicting the workflow of IP-MS. (**b**) Table showing the candidates of UCHL1 interactions, the number of PSMs > 20. (**c**) Co-IP of UCHL1 protein with GRP78 protein in CFs stimulated with TGF-β1. (**d**) Immunofluorescence staining showing the co-localisation of UCHL1 with GRP78 in sham and MI hearts. Scale bar = 200 μm. (**e**) Immunofluorescence staining showing the co-localisation of UCHL1 with GRP78 in CFs with/without TGF-β1 stimulation. Scale bar = 200 μm.

To identify the underlying mechanisms of UCHL1 on post-MI fibrosis, we performed immunoprecipitation-mass spectrometer (IP-MS) in TGF-β1-induced CFs to find UCHL1 interactors (Fig. 4a). Through analysing the MS data, we list the proteins which number of PSMs > 20 as candidates that IP with UCHL1(Fig. 4b). GRP78, also named endoplasmic reticulum chaperone BiP, was ranked at the top of the list (Fig. 4b). Therefore, we detected if there was crosstalk between UCHL1 and GRP78. We found that UCHL1 interacted with GRP78 in CFs with TGF-β1 stimulation using Co-IP (Fig. 4c). Immunofluorescence staining showed that UCHL1 colocalised with GRP78 in fibrotic areas of MI hearts (Fig. 4d) as well as in CFs induced with/without TGF-β1 (Fig. 4e).

**Figure 5:**
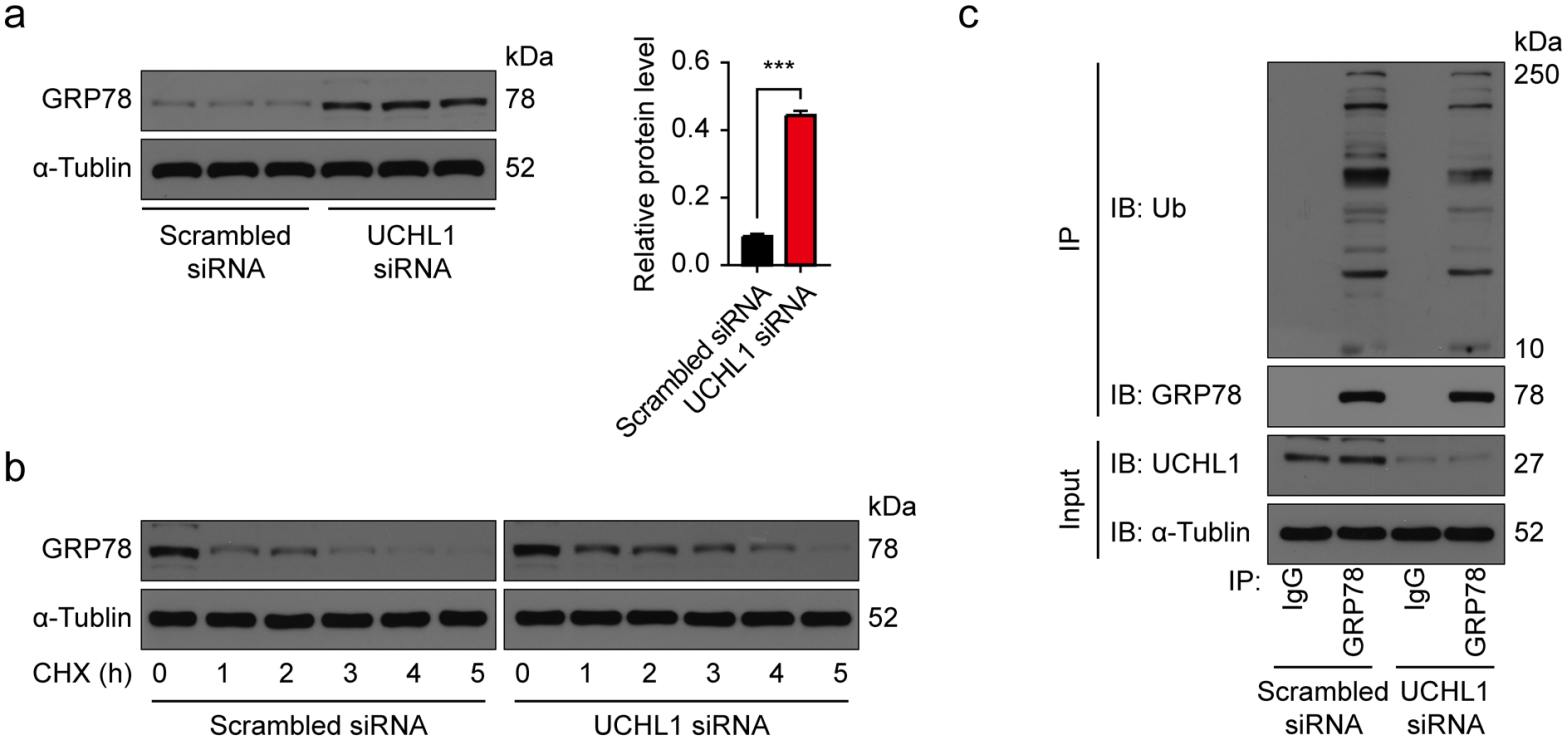
UCHL1 degrades GRP78 by enhancing levels of ubiquitination. (**a**) Western blot and average data showing GRP78 protein levels in CFs with/without UCHL1 knockdown. n = 3, ***P < 0.001. (**b**) Western blot showing the protein levels of GRP78 in CFs with/without UCHL1 knockdown treated with 20 μM CHX at the indicated times. (**c**) IP and Western blot showing the ubiquitination level of GRP78 protein in TGF-β1 stimulated CFs with/without UCHL1 knockdown.

To find whether GRP78 is regulated by UCHL1, we examined the GRP78 protein level and found that knockdown of UCHL1 in CFs elevates the GRP78 protein levels (Fig. 5a). We next blocked protein synthesis in CFs with/without UCHL1 knockdown using CHX for 0–5 h and measured the subsequent GRP78 degradation. We found that the rate of GRP78 degradation was significantly decreased with UCHL1 knock down (Fig. 5b). Downregulation of UCHL1 by UCHL1 siRNA decreased poly-ubiquitination of GRP78 (Fig. 5c). Taken together, these results suggest that UCHL1 interacts with GRP78 and downregulates it via ubiquitination.

**Figure 6:**
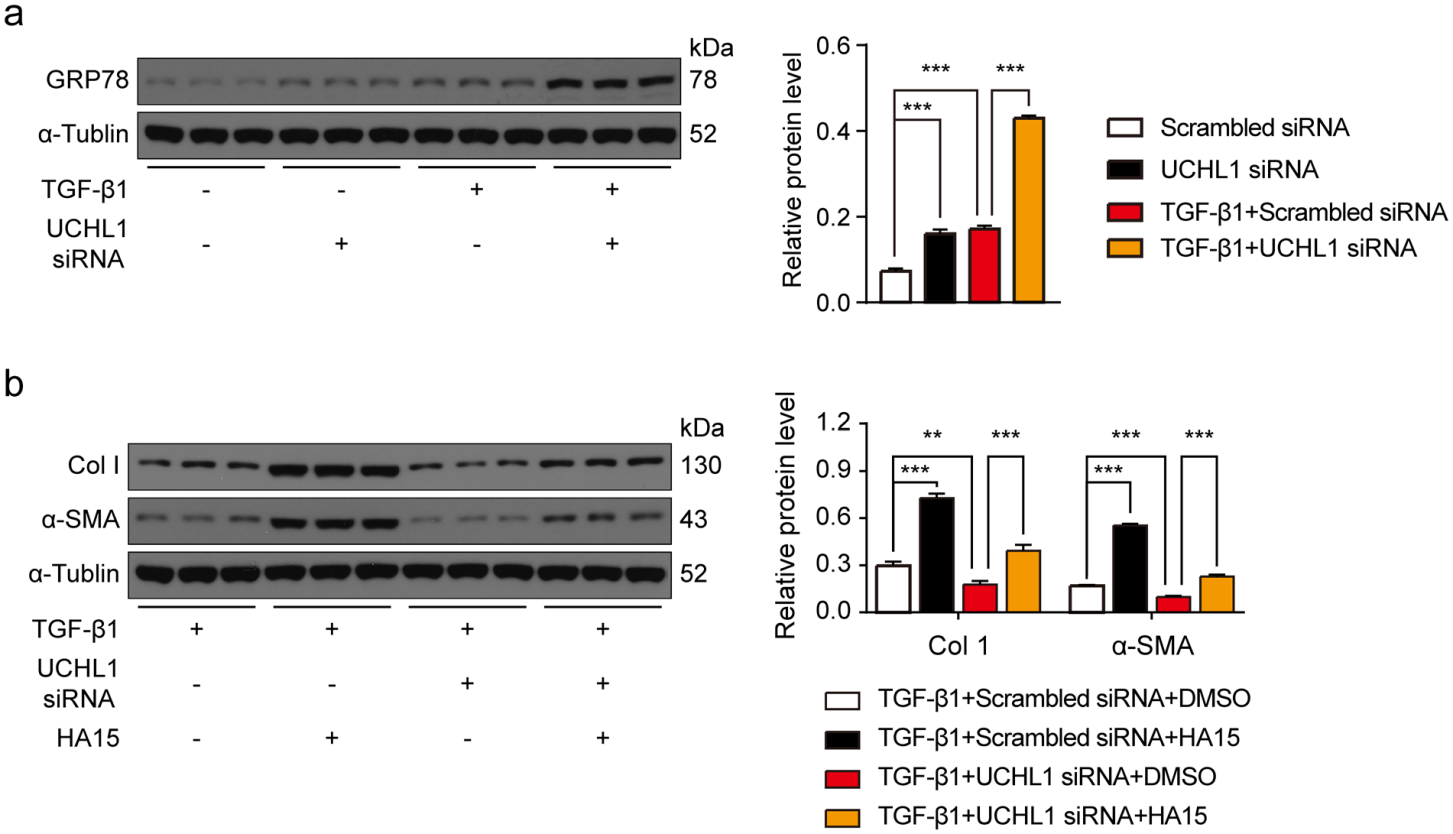
Inhibition of GRP78 diminished the inhibitory effect of UCHL1 knockdown on TGF-β1-mediated fibrotic responses in CFs. (**a**) Western blot and average data showing the protein level of GRP78 in CFs with/without UCHL1 knockdown with/without TGF-β1 stimulation. n = 3, ***P < 0.001. (**b**) Western blot and average data showing levels of profibrogenic proteins Col1 and α-SMA in TGF-β1 stimulated CFs with/without UCHL1 knockdown treated with/without GRP78 inhibitor HA15. n = 3, **P < 0.01, ***P < 0.001.

**Figure 7:**
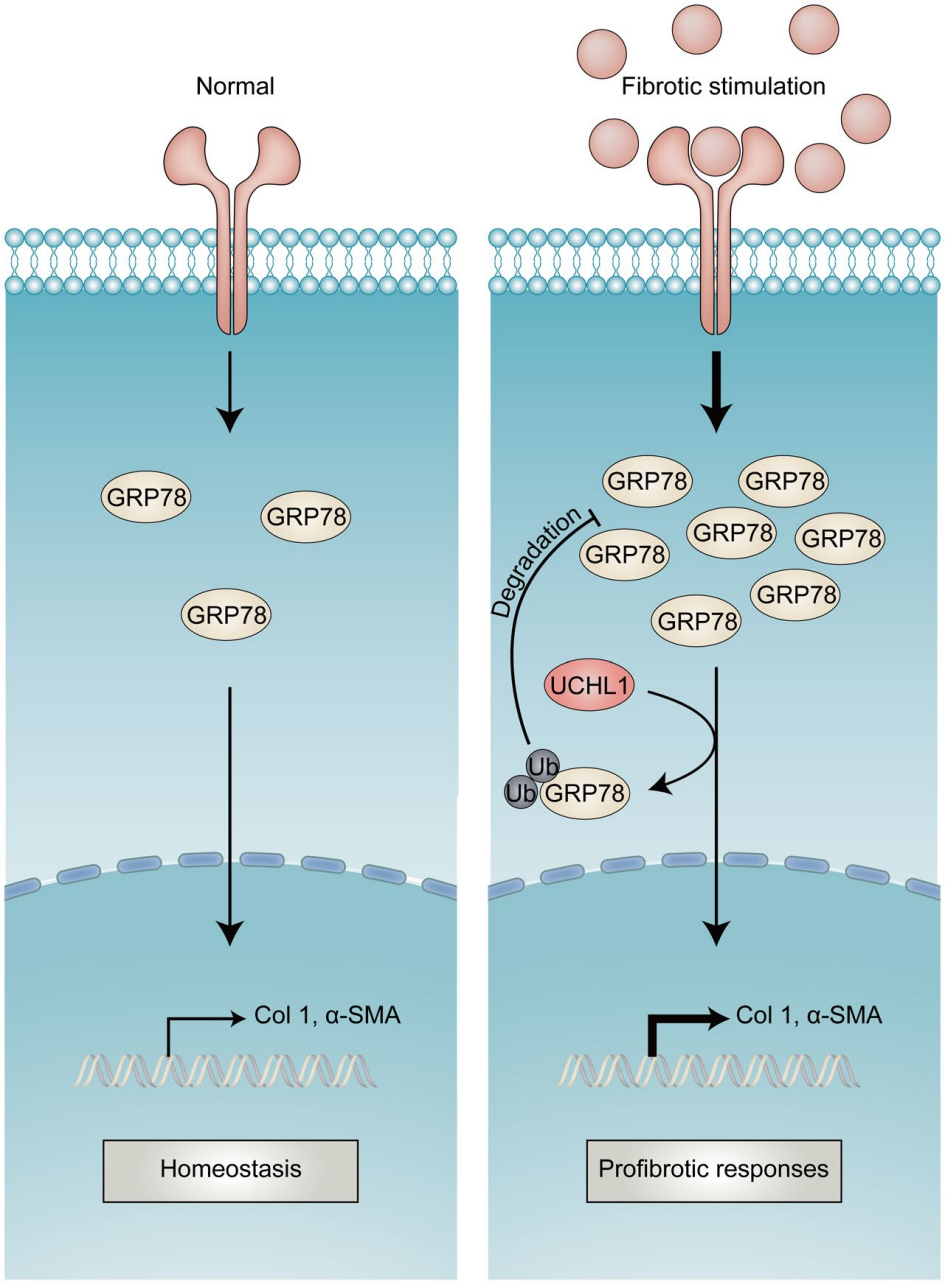
Schematic diagram illustrating the regulation of UCHL1 on GRP78 in post-MI fibrosis.

Moreover, we wondered if the effect of UCHL1 on fibrosis was related to GRP78, so we determined the GRP78 protein levels in CFs with/without TGF-β1 and treated with/without UCHL1 knockdown. We found that GRP78 protein levels were upregulated by TGF-β1 and knockdown of UCHL1 led to greater increases in GRP78 levels (Fig. 6a). We also used HA15, a GRP78 inhibitor, to inhibit GRP78, and found that inhibition of GRP78 abolished the downregulation of Col1 and α-SMA in response to UCHL1 siRNA in CFs with TGF-β1 stimulation (Fig. 6b). Together, our results suggest that UCHL1 regulates cardiac fibrosis by GRP78 (Fig. 7).

## Discussion

Cardiac fibrosis is a critical pathologic process in ventricular remodelling after MI. Since the role of UPS in mediating cardiac fibrosis is unknown, we sought to determine the role of UCHL1 in cardiac fibrosis following MI. UCHL1 inhibition attenuated cardiac dysfunction and fibrosis following MI. LDN and UCHL1 siRNA inactivated CFs and notably, GRP78, a critical molecule involved in cellular homeostasis by handling unfolded proteins, was identified as an interactor of UCHL1. UCHL1 prompted GRP78 degradation by interacting with and ubiquitinating it. Knockdown of UCHL1 upregulated GRP78 in CFs with TGF-β1 stimulation and inhibition of GRP78 diminished the antifibrotic effect of UCHL1 knockdown. Thus, the UCHL1-GRP78 axis could be a novel target for cardiac fibrosis therapy.

Dobaczewski et al*.* found that UCHL1 was dramatically elevated at 7 days and lasted for at least 21 days post but the underlying mechanism behind this process was unclear^26^. We hypothesised that UCHL1 may be a key mediator of post-MI remodelling. Since no studies focus on the role of UCHL1 in MI, we aimed to investigate the UCHL1 on mouse MI model. We inhibited UCHL1 using active site-directed inhibitor LDN^30^, and found that the treatment improved the cardiac function and attenuated cardiac fibrosis after MI. UCHL1 staining was observed in the area of fibrosis in the infarct heart using IHC. Therefore, we aimed to assess the negative effect of UCHL1 on the infarct heart. As expected, we verified the antifibrotic role of UCHL1 inhibition on CFs stimulated with TGF-β1 using LDN. Results from previous studies had very different results that suggest that UCHL1 is a promising repressor for CF activation^31^. The differences between these studies may be due to the source of CFs, as the CFs of our study are isolated from adult mice rather than neonatal rats; the neonatal heart but not the adult heart, possesses regeneration potential. Another potential difference between the studies is that our study stimulated CFs with TGF-β1, while previous studies used PDGF. The role of UCHL1 relies on the context of the cells. The antifibrotic role of LDN on the heart was also shown by another study that examined atrial fibrillation but did not use cell culture models^27^. In addition, the pro-activation effect of UCHL1 is observed in other types of fibroblasts, such as cancer-associated fibroblasts and hepatic stellate cells^29,32^. These findings suggest a novel potential target in CF activation.

To find the underlying mechanisms of UCHL1, we screened its interactor using IP-MS and identified GRP78 as candidate interactors. This is consistent with the finding that GRP78 is colocalised with UCHL1 in COS-7 cells^33^. Thus, there exists a possibility that UCHL1 interacts with GRP78 through the UCHL1-GRP78 complex. GRP78 is a molecular chaperone of the Hsp70 family with protective properties, such as stabilising the calcium concentration of endoplasmic reticulum as a calcium binding protein, transferring the misfolded protein out of the endoplasmic reticulum and helping to fold unfolded proteins^34^. To pinpoint if there is a direct interaction between UCHL1 and GRP78, we validated the interaction of UCHL1 and GRP78 via co-immunofluorescence and co-immunoprecipitation. We found that GRP78 was significantly increased in CFs treated with UCHL1 siRNA, consistent with an investigation in SK-N-SH cells^35^. The upregulation of GRP78 resulted from the reduction of ubiquitination by UCHL1 knockdown. Therefore, the effect of UCHL1 on cardiac fibrosis may be due to its control of GRP78.

GRP78 is a master mediator of the unfolded protein response^34^. The effect of GRP78 on fibrosis is partly embodied in the ‘two-edged sword’ function of the unfolded protein response in fibrosis-related pulmonary diseases and diabetic nephropathy^36-38^. When it comes to fibrosis in MI, the role of GRP78 on ischaemic myocardium, either protective or harmful, lies on environment^39^. We found that GRP78 was upregulated in TGF-β1 stimulated CFs and a greater increase of GRP78 was observed in TGF-β1 stimulated CFs treated with UCHL1 siRNA. So GRP78 may play a protective role in TGF-β1 stimulated CFs. To find out whether UCHL1 exerts its pro-fibrosis effect through inhibition of the protective effect of GRP78 in the process of cardiac fibrosis, we used HA15 to inhibit the GRP78. HA15 specifically targets GRP78 and inhibits its ATPase activity^40^. HA15 diminishes the antifibrotic effect of UCHL1 siRNA with TGF-β1 stimulation. Collectively, our data suggests that the UCHL1-GRP78 complex plays a role in the regulation of cardiac fibrosis.

One limitation of this study is the gene manipulation. UCHL1 knockout mice should be used in future studies to confirm the effect of UCHL1 knockout. As UCHL1 was also overexpressed in cardiomyocytes, and the effect of UCHL1 on cardiomyocytes remains to be determined^26^. The signalling pathways upstream of UCHL1 remain unknown, which may relate to osteopontin which would link CFs and cardiomyocytes^41^. One potential challenge for clinical translation is that a clinical therapeutic would need to be tissue specific, and have an inhibitory effect on UCHL1 solely in the heart.

This study demonstrates that UCHL1 enhances cardiac fibrosis after MI by interacting with and downregulating GRP78 by ubiquitination (Fig. 7). Interventions that target the UCHL1-GRP78 interaction may be a potential therapeutic strategy against cardiac fibrosis post-MI. Our findings deepen the understanding of maladaptive fibrogenesis post-MI with a focus on UPS.

## Methods

### Induction of MI and in vivo experimental design

MI was induced via permanent ligation of the left anterior descending coronary artery (LAD). Briefly, mice were anaesthetised with sodium pentobarbital (intraperitoneal injection, 50 mg/kg; Merck, China) and mechanically ventilated by the HX-101E ventilator (Chengdu Taimeng Software Ltd., China). The thoracotomy was performed in the 4th left intercostal space, and then the LAD was occluded with 7–0 polyester sutures. The chest wall was closed using 5–0 polyester sutures. The success of the operation was confirmed by blanched heart apex and elevated ST segment on the ECG. Sham-operation was performed in the same manner just leaving the LAD without an occlusion.

UCHL1 protein levels were measured in the heart at 1 day, 7 days and 14 days after operation. The surviving sham mice (n = 20) were sacrificed separately at 1 day, 7 days and 14 days after operation (n = 6, for each time). The surviving MI mice (n = 22) were sacrificed separately at 1 day, 7 days and 14 days after operation (n = 6, for each time). For histological analyses, three hearts of mice from each time point were obtained and processed for immunohistochemistry. For protein level assessment, three hearts from each time point were obtained and processed for western blot.

Sham/MI mice were administrated with/without LDN57444 (LDN) at a concentration of 0.4 mg/kg/days (ip.) for 14 days from 1 day after operation. The surviving sham mice were randomly divided into 2 groups (n = 10 in each group): (1) Sham + DMSO group (treated with DMSO in saline for 14 days, i.p.); (2) Sham + LDN group (treated with LDN, 0.4 mg/kg/days, for 14 days, i.p.). The surviving MI mice were randomly divided into 2 groups (n = 12 in each group) (1) MI + DMSO group (treated with DMSO in saline for 14 days, i.p.), and (2) MI + LDN group (treated with LDN, 0.4 mg/kg/days, for 14 days, i.p.). The remaining mice were evaluated for cardiac function and sacrificed 14 days after operation. For protein level assessment, six hearts of mice from different groups were obtained and processed for western blot. For histological analyses, three hearts of mice from different groups were obtained and processed for immunohistochemistry and Masson's trichrome staining.

### Echocardiography

Echocardiography was performed 14 days after operation using a Vevo 1,100 High Resolution Ultrasonic Imaging System (Visualsonics, Toronto, Canada). The mice were slightly anaesthetised with pentobarbital sodium (20 mg/kg) and kept at a heart rate > 400 bpm under an ECG monitor. The hearts’ long and short axis views were obtained in B-mode and M-mode, respectively. The M-mode photographs were acquired between the two papillary muscles. Left ventricular EF and FS were automatically calculated by the echocardiography software. All measurements were the mean of three successive cardiac cycles.

### Histological analyses

While mice were under deep anaesthesia, mouse hearts were immediately extracted and fixed in 4% formaldehyde. Then the fixed hearts sequentially underwent dehydration and paraffin embedding. Subsequently, 4 μm cross-sectional slices were obtained.

#### Immunohistochemistry (IHC)

After blocking with 10% serum of the secondary antibody host for 1 h, the slices were incubated with a primary antibody against UCHL1 (1:500, Proteintech, 14730-1-AP) overnight at 4 °C. The slices were then incubated with streptavidin-HRP conjugated secondary antibody for 1 h at room temperature. A drop of 3,3′-diaminobenzidine (DAB) solution (Boster, AR1022, China) was applied until the brown positive stain appeared. Then the counterstain was performed with haematoxylin. All the slices were scanned with an Aperio VERSA System (Leica Biosystems, Germany). Three representative images from each sample were taken and analysed using the Image J software (National Institutes of Health, Bethesda, USA).

#### Masson's trichrome staining

To evaluate cardiac fibrosis, Masson's trichrome staining was performed in paraffin-embedded heart cross-sectional slices at the mid-papillary level. The area stained blue was the fibrosis area. The slices were captured using an Aperio VERSA System (Leica Biosystems, Germany). The areas were measured using the Image J software (National Institutes of Health, USA). The ratio of fibrosis to the whole left ventricle was used to compute infarct size expressed as a percentage of the left ventricle. And the ratio of fibrosis to viable myocardium in the peri-infarct area was used to compute interstitial fibrosis expressed as a percentage of surface area^42^.

### Adult mouse CF isolation

CFs were isolated from 4-month-old male C57BL/6 mice that weighed 20–25 g by collagenase digestion. Briefly, after deep anaesthesia was induced in mice, the cardiac ventricles were rapidly removed and rinsed with cold sterile PBS under sterile conditions. Next, the ventricles were finely minced followed by digestion in 10 ml DMEM containing 0.2% type 2 collagenase (Worthington Biochemical, Lakewood, NJ, USA) at 37 °C for 90 min with gentle shaking. The supernatant was then centrifuged at 1000* g* for 5 min and resuspended in culture medium (DMEM containing 10% FBS supplemented with 1% penicillin and 1% streptomycin) before plating into 10 cm culture dishes. Non-adherent cells were removed after culturing for 4 h and the adherent cells were further cultured in fresh culture medium. Afterwards, the isolated cells were detected by immunofluorescence using antibodies against vimentin. CFs, which are positive for vimentin, accounted for > 95% (Supplemental Fig. 2). The isolated cells were used for in vitro studies between passage 2 and 4.

### UCHL1 siRNA transection

Knockdown of UCHL1 was conducted by transfecting siRNA. Briefly, before transfection, CFs were dissociated and resuspended at a cellular density of 1 × 10^5^/ml. Cell suspensions were transfected with siRNA at a final siRNA concentration of 10 nM specifically against UCHL1 (sense, 5′-aacacttggctctatcttcggtt-3′) or scrambled siRNA (sense, 5′-tgcccaaattcatcgtctgtgtt-3′) using Lipofectamine 2000 (Invitrogen, Carlsbad, CA, USA) for 48 h. The efficiency of transfection was evaluated by western blot.

### In vitro chemical treatment

CFs with/without UCHL1 knockdown were incubated in serum-free DMEM medium for 24 h and subsequently stimulated with 10 ng/ml of TGF-β1 in the absence of serum for another 24 h, to mimic post-MI conditions. To inhibit UCHL1 activity, 5 μM of LDN (HY-18637; MedChemExpress) was added for 6 h before the completion of the TGF-β1 stimulation. The GRP78 inhibitor HA15 (HY-100437, MedChemExpress), at a final concentration of 10 μM, was treated for 24 h at the beginning of TGF-β1 stimulation.

### Cycloheximide (CHX) chase assay

After transfection, CFs were incubated with CHX (20 µM, HY-12320, MedChemExpress), then harvested at 0, 1, 2, 3, 4 and 5 h after CHX addition. The lysates were subjected to immunoblot for evaluation of GRP78 protein stability.

### Western blot

Protein was extracted from heart tissue or CFs using RIPA buffer (Beyotime, China) containing protease inhibitor (Complete Mini EDTA-free, Roche) and phosphatase inhibitor (PhosSTOP, Roche). The concentration was quantified using a BCA Kit (Beyotime, China). Equal protein (50–100 μg) was subjected to 6–12% SDS-PAGE electrophoresis and transferred to nitrocellulose (NC) membranes. After blocking with QuickBlock Blocking Buffer (Beyotime, P0252-500 ml, China) for 30 min, the NC membranes were incubated with primary antibodies against UCHL1 (1:2,000, Proteintech, 14730-1-AP), UCHL3 (1:1,000, Proteintech, 12384-1-AP), Collagen I (1:500, Abcam, ab138492), α-SMA (1:1,000, Proteintech, 14395-1-AP), GRP78 (1:1,000, Proteintech, 11587-1-AP), Ubiquitin (1:1,000, Abcam, ab140601) and α-Tubulin (1:1,000, Proteintech, 11224-1-AP). NC membranes were incubated with the respective HRP-conjugated secondary antibodies (Boster, BA1054/BA1050) and visualised with a ChemiDoc Touch Imaging System (Bio-Rad, USA). Specific bands were analysed with Image J (Version 1.4.3.67).

### Co-immunoprecipitation (Co-IP)

We conducted the Co-IP with Pierce Classic Magnetic IP/Co-IP Kit (Thermo Fischer Scientific, 88804, USA) according to the manufacturer’s instructions. Briefly, samples were extracted from CFs (10 cm dish per sample) using lysis buffer. The samples were incubated with rabbit UCHL1 antibody (1:100, Proteintech, 14730-1-AP), rabbit GRP78 antibody (1:100, Proteintech, 11587-1-AP, China) or rabbit isotype control IgG (1:250, Abclonal, AC005, China) with agitated rotation at 4 °C overnight. 25 μl protein A/G magnetic beads were added to the samples and they were incubated for 4 h at 4 °C. The beads were washed three times with lysis buffer to remove un-bound proteins and the bound proteins were eluted using 20 μl elution buffer and shaking for 10 min at room temperature. Then, mass spectrometry or western blotting was performed using the eluted proteins.

### Immunoprecipitation-mass spectrometer (IP-MS)

The immunoprecipitants of eluted proteins were subjected SDS-PAGE. Gel lanes were excised following coomassie blue staining and submitted for mass spectrometry with Orbitrap Elite mass spectrometers (Thermo Fisher Scientific).

### Immunofluorescence staining

Upon blocking in QuickBlock Blocking Buffer (Beyotime, Cat: P0260, Shanghai, China) for 1 h at room temperature, the slices (seeded with CFs or embedded with heart tissue) were incubated with primary antibodies against α-SMA (1:100, Proteintech, 14395-1-AP), UCHL1 (1:200, Proteintech, China), and GRP78 (1:200, Proteintech, China) at 4 °C overnight. Thereafter, secondary antibodies (goat-anti-rabbit Alexa Fluor 555-conjugated antibody, Cell Signaling Technology; goat-anti-rabbit Alexa Fluor 488-conjugated antibody, Cell Signaling Technology) were applied at room temperature for 1 h for fluorescent labelling. Nuclei were counterstained with DAPI (1:2000, Sigma-Aldrich, USA). All images were captured with a confocal microscopy (LSM800, Zeiss, Germany). Three images of each simple were used for quantification.

### Ubiquitination assay

CFs were treated with Scrambled siRNA or UCHL1 siRNA for 48 h. MG132 (10 μM) was added 12 h before the cells were harvested. Whole cell lysates were immunoprecipitated with anti-GRP78 antibody and then immunoblotted with anti-ubiquitin (Ub) antibody to evaluate the ubiquitination level of GRP78 protein.

### Statistical analysis

Statistical analysis was performed using GraphPad Prism 6 (GraphPad Software Inc, USA). Differences between two experimental groups was analysed using the Student’s *t*-test and multiple comparison groups were analysed using one-way ANOVA with Tukey test. All data were presented as the means ± SD. *P* < 0.05 was considered statistically significant.

### Study approval

All animal experiments and surgical procedures were conducted in line with the Animal Care and Use Committee Guide of Wuhan University, which conforms to the Guide for the Care and Use of Laboratory Animals of the National Institutes of Health (NIH Publication No. 85-23, revised 1996). All methods were performed in accordance with relevant guidelines and regulations.

## Supplementary information

Supplementary information.

## Data Availability

All relevant data supporting the conclusions are included in this published article and its supplementary information files.
