# Supplementary material for: Ubiquitin C-terminal hydrolase L1 (UCHL1) regulates post-myocardial infarction cardiac fibrosis through glucose-regulated protein of 78 kDa (GRP78)
Source: Sci Rep. 2020 Jun 30;10:10604. doi: 10.1038/s41598-020-67746-4 (PMC7326919; doi:10.1038/s41598-020-67746-4)
Supplement: Supplementary file 1 — Supplementary information. [file 41598_2020_67746_MOESM1_ESM.pdf]

SUPPLEMENTARY INFORMATION

Ubiquitin C-terminal hydrolase L1 (UCHL1) regulates post-myocardial infarction cardiac fibrosis through glucose-regulated protein of 78 kDa (GRP78)

Qian Lei[1], Tao Yi[3], Hang Li[1], Zhijie Yan[1], Zhan Lv[1], Gerui Li[1], Yanggan Wang[1, 2*]

[1]Department of Internal Medicine, Zhongnan Hospital of Wuhan University, Wuhan University, Wuhan 4300/1, China

[2]Medical Research Institute of Wuhan University, Wuhan University, Wuhan, China

[3]Department of Cardiology, Zhongsan People’s Hospital, Zhongsan, China

[*]Corresponding author. E-mail address: Wangyg-wuhan@outlook.com

Contributions: QL designed the study and wrote the article. TY collected and analysed the data. HL, ZJY, ZL, and GRL provided technical support. YGW revised the article.

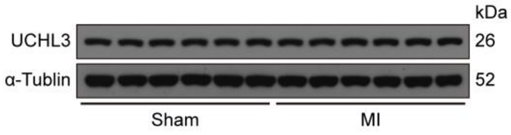

Supplemental Fig. 1 Western blot and average data showing UCHL3 protein level in hearts of Sham/MI group. n = 6.

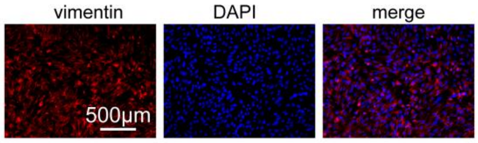

Supplemental Fig. 2 Immunofluorescence staining of vimentin in CFs.
